# Multifunctional Carbon Aerogels Derived by Sol–Gel Process of Natural Polysaccharides of Different Botanical Origin

**DOI:** 10.3390/ma10111336

**Published:** 2017-11-21

**Authors:** Monika Bakierska, Agnieszka Chojnacka, Michał Świętosławski, Piotr Natkański, Marta Gajewska, Małgorzata Rutkowska, Marcin Molenda

**Affiliations:** 1 Faculty of Chemistry, Jagiellonian University, Gronostajowa 2, 30-387 Krakow, Poland; agn.chojn@gmail.com (A.C.); m.swietoslawski@gmail.com (M.Ś.); natkansk@chemia.uj.edu.pl (P.N.); rutkowsm@chemia.uj.edu.pl (M.R.); 2 Academic Centre for Materials and Nanotechnology, AGH University of Science and Technology, Mickiewicza 30, 30-059 Krakow, Poland; marta.gajewska@agh.edu.pl

**Keywords:** polysaccharide, starch, sol–gel polycondensation, ambient pressure drying, carbon aerogel, porous carbon nanomaterial, electrical conductivity

## Abstract

In this manuscript, we describe the results of our recent studies on carbon aerogels derived from natural starches. A facile method for the fabrication of carbon aerogels is presented. Moreover, the complete analysis of the carbonization process of different starch aerogels (potato, maize, and rice) was performed using thermogravimetric studies combined with a detailed analysis of evolved decomposition products. The prepared carbon aerogels were studied in terms of their morphology and electrical properties to relate the origin of starch precursor with final properties of carbon materials. The obtained results confirmed the differences in carbon aerogels’ morphology, especially in materials’ specific surface areas, depending on the botanical origin of precursors. The electrical conductivity measurements suggest that carbon aerogels with the best electrical properties can be obtained from potato starch.

## 1. Introduction

It is often assumed that aerogels are products of the latest technology. As a matter of fact, the first aerogels were prepared in 1931 by Steven S. Kistler [1,2]. After this discovery, new developments in aerogels science and technology occurred rapidly.

Carbon aerogels are a special class of nanostructured and highly porous aerogels that have been known for about 28 years, and were prepared for the first time by the American scientist Richard W. Pekala and his colleagues at Lawrence Livermore National Laboratory [3]. In contrast to the conventional porous carbon materials, carbon aerogels have a continuous network structure of interconnected nanosized primary particles [4]. Furthermore, these ultralight carbon materials exhibit extraordinary properties, including well-defined and controlled porosity, large surface area, chemical stability, and low electrical resistance, which make them desirable materials for a wide range of technological applications (e.g., thermal insulation [5,6,7], heavy metal or organic solvents absorption [8,9,10], energy storage [11,12,13,14], catalyst supports [15,16,17], and biomedicine [18,19,20]).

Generally, carbon aerogels are formed from the sol–gel polymerization of resorcinol and formaldehyde, followed by drying and subsequent pyrolysis at an elevated temperature in an inert atmosphere. The resorcinol-formaldehyde aerogels (RF) are produced in a way similar to silica aerogels. Resorcinol reacts quickly with formaldehyde to form numerous hydroxymethyl-substituted species. These species condense into surface of functionalized polymer “clusters” that crosslink to form a gel [21]. One major challenge in the preparation of aerogels is to eliminate the liquid solvent from the gel without collapsing the already existing nanoporous structure, thereby avoiding the subsequent shrinkage and cracking of the dried gel. Currently, to overcome these obstacles, supercritical drying process assisted by the use of supercritical fluids (usually CO_2_) is used [22]. In the literature, some reports on other drying techniques such as freeze drying [23] and traditional drying procedures under ambient pressure [24,25] can be found as well.

As already mentioned, the main source of carbon aerogels are resorcinol-formaldehyde aerogels; however, it was shown that part of the resorcinol used for the synthesis of the RF aerogel may be replaced by cresols [26]. The precursor of the organic aerogels may also include: melamine [27], isocyanate [28], polyvinyl chloride [29], phenol [30], furfural [31], and many other compounds. These synthetic materials are often toxic and expensive. That is why new alternatives are investigated. The use of natural polysaccharides and their derivatives is considered to be more appealing owing to their abundance, availability, renewability, stability, non-toxicity, and low cost [18,19,24,25]. Of particular interest is the use of starch that is common with a large variation depending on the botanical origin. It consists of two glucose polymers: amylose (linear polymer) and amylopectin (branched polymer). Starch separated from various plant materials include different amounts of amylose and amylopectin. The length of polysaccharides chain also varies depending on the botanical origin of the starch. These variables in the composition of starch affect its structure and properties [32]. Hence, it can be concluded, that they will also affect the properties arising from the synthesized carbon materials.

In this work, the impact of the botanical origin of natural starches on thermal, structural, and electrical characteristics as well as morphology of carbon aerogels, obtained at different temperatures, was determined.

## 2. Materials and Methods

Carbon aerogels (CAGs) based on different types of starch (potato, maize, and rice) were obtained by the carbonization of organic aerogels. Precursors of carbon aerogels were prepared via the sol–gel polycondensation process according to the procedure described in our previous paper [25]. Briefly, starches of potato, maize, and rice origins (Sigma-Aldrich, Saint Louis, MO, USA) were dispersed in water with appropriate dilution ratio (the concentration of the solutions for potato and rice starches was 10 wt %, for maize starch it was 15 wt %). Suspensions of starches were stirred and heated up to the gelatinization temperature. Subsequently, the solvent exchange by immersing the aqueous gels for 12 days in the ethanol (96%, Avantor Performance Materials - formerly POCH S.A., Gliwice, Poland) was carried out. Afterwards, the alcogels were dried under ambient pressure in air at 50 °C for 1 day. As-obtained organic aerogels (OAGs) based on potato (OAGPS), maize (OAGMS) and rice starch (OAGRS) were analyzed in the context of thermal decomposition and morphology characteristics. Starch aerogels were then pyrolysed under argon flow (purity 99.999%, 50 mL∙min^−1^, Air Products, Allentown, PA, USA) at 700 °C, 800 °C, and 900 °C for 6 h, which allowed carbon aerogels (so called CAGPS, CAGMS, CAGRS, consequently) to be obtained. At this point, it should be mentioned that the OAGs for carbonization were prepared as coarse powders and CAGs after the heat treatment remained in this form. However, for electrochemical application, the CAGs need to be ground into uniform fine powders, and the samples in this form are presented in the following paper. That is why the grinding of CAGs samples after pyrolysis was performed in an agate mortar for about 30 min for each sample.

The thermal decomposition of organic aerogels was studied by means of thermogravimetric analysis coupled with evolved gas analysis with infrared spectroscopy detection (EGA(FTIR)-TGA/DTA/DTG method). The experiments were carried out using SDT Q600 thermobalance (TA Instruments, New Castle, DE, USA) coupled with a Fourier transform infrared (FTIR) spectrometer (Nicolet 6700 FTIR, Thermo Fisher Scientific, Waltham, MA, USA) by FTIR-TGA interface (Thermo Fisher Scientific, Waltham, MA, USA). The measurements were performed in an inert gas flow (N_2_, 20 mL∙min^−1^) for samples with the weight of 20 mg placed in a corundum crucible, in the temperature range of 20–1000 °C and at a heating rate equal to 5 °C∙min^−1^. The 2D and 3D FTIR spectral maps of evolved gaseous products were recorded with resolution of 4 cm^−1^ collecting eight scans for each spectrum. The morphology of the materials was characterized using an FEI Versa 3D (FEG—Field Emission Gun) scanning electron microscope (FEI Company, Hillsboro, OR, USA). The crystal structure of the carbon aerogels was characterized by powder X-ray diffraction (XRD) using BRUKER D2 PHASER (Billerica, MA, USA). The Cu Kα radiation (λ=0.154184 nm) in the range of 10–60° (2θ) with a step of 0.02° was used. To determine the amount (a weight percent) of carbon, hydrogen, and nitrogen elements in the obtained carbon compounds, the elemental analysis (CHN analysis) was conducted using micro analyzer vario MICRO cube coupled with microbalance (Elementar, Langenselbold, Germany). Before the CHN determination, the CAG samples were dried in vacuum oven under 80 mbar for 3 h at 80 °C. The evaluation of chemical composition was performed with an accuracy of 0.3%. The electrical conductivity (EC) studies were carried out using semi-4-probe method with 1 mA alternating current (at a fixed frequency of 33 Hz) within temperature range from −20 to +40 °C by means of state of the art Sigma1 apparatus. The powder samples (with a thickness of about 2.5 mm) were placed in a glass tube between the parallel flat and gold circular electrodes (with 5 mm in diameter) and pressed by an electrode piston until the measured resistance of the samples remained constant and appropriate electrical contact was assured. Porous features of the resulting samples were evaluated from N_2_ sorption at −196 °C measured with 3Flex v1.00 automated gas adsorption system (Micromeritics, Norcross, GA, USA). Before the analysis, the samples were degassed under vacuum at 350 °C for 24 h. The specific surface area (S_BET_) was determined by the single point surface area at $\frac{p}{p_{0}}=0.2$.

## 3. Results and Discussion

**Figure 1:**
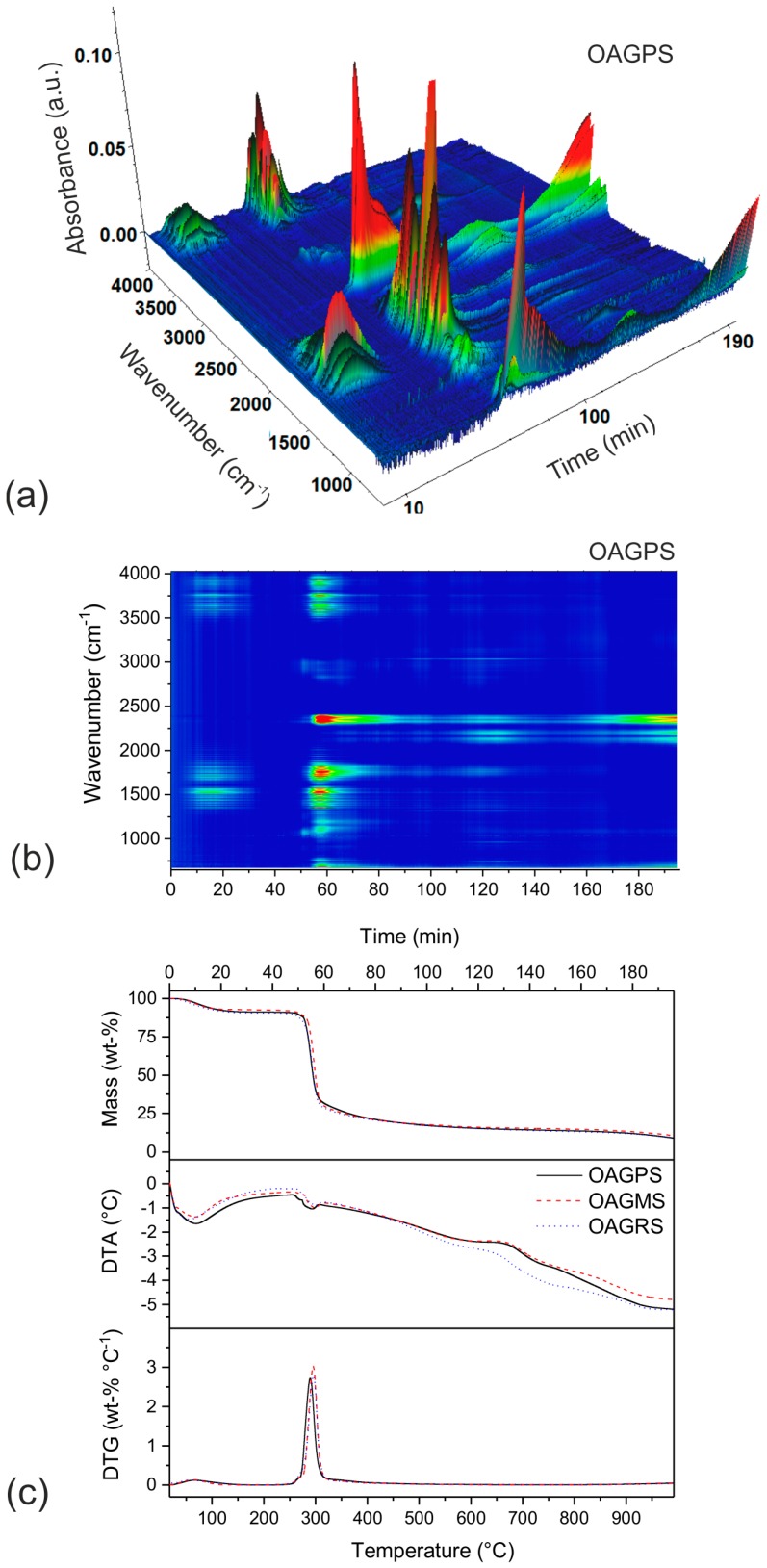
3D and 2D Fourier transform infrared (FTIR) spectrum maps of gaseous products evolved during thermal decomposition of (**a**,**b**) the organic aerogel sample based on potato starch (OAGPS) and (**c**) TGA/DTA/DTG profiles for OAGPS, OAGMS (OAG based on maize starch) and OAGRS (OAG based on rice starch) materials.

The thermal decomposition of various origin starches carried out under inert atmosphere was studied with the use of the EGA(FTIR)-TGA/DTA/DTG coupled method. The recorded 3D and 2D FTIR spectral maps of gaseous decomposition products created during heating of OAGPS sample (as an example) as well as TGA/DTA/DTG curves for all of the examined materials (OAGPS, OAGMS, OAGRS) are presented in Figure 1.

**Figure 2:**
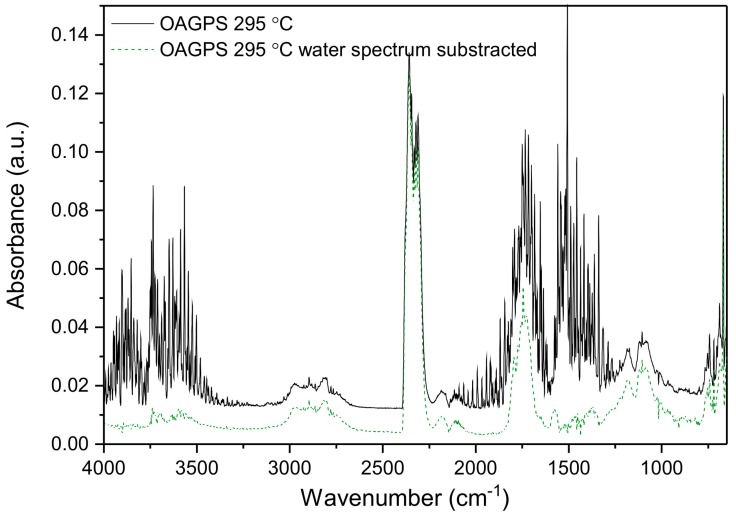
FTIR spectrum recorded at 295 °C for OAGPS sample.

According to the obtained results, the pyrolysis process for all types of starch is similar. At the temperatures below 230 °C, only the vaporization of water from material is observed, which is confirmed by the presence of characteristic bands at 4000–3500 cm^−1^ and 2000–1300 cm^−1^ in the FTIR maps. The content of moisture in the studied samples was between 8.8 and 9.5 wt %. The main starch decomposition stage ran within the temperature range of 230–350 °C, connected with additional mass loss equal to 64.7 wt % (for OAGPS), 66.9 wt % (for OAGMS), and 65.6 wt % (for OAGRS). For more detailed identification of gaseous products, the FTIR spectrum recorded at 295 °C (the temperature corresponding to DTG maximum) is shown in Figure 2.

In order to analyze the carbonyl compound, the water vapor spectrum was subtracted from the above-mentioned one. Based on the results illustrated in Figure 2, the main products of anaerobic thermal decomposition of starch in this temperature range are CO_2_ (3760–3580 cm^−1^, 2390–2290 cm^−1^, and 720–650 cm^−1^), H_2_O (4000–3500 cm^−1^, 2000–1300 cm^−1^) and small amounts of CO (2220–2080 cm^−1^). Moreover, the presence of organic gaseous products is clearly observed. Among the bands which appeared, we can distinguish signals coming from stretching vibrations of C–H in aliphatic groups (bands at 2970 cm^−1^ and 2895 cm^−1^), stretching vibrations C–H in O=C–H groups (band at 2810 cm^−1^), stretching vibrations of C=O (bands at 1795 cm^−1^ and 1740 cm^−1^), C=C in aromatic rings (1650 cm^−1^, 1575 cm^−1^), deformation vibrations of C–H in aliphatic groups (bands in the range of 1300–1500 cm^−1^), and C–O stretching of alcohol and carbohydrate functional groups (bands at 1181 cm^−1^, 1108 cm^−1^) [33,34,35]. The listed absorption peaks correspond to the presence of carbonyl compounds, unsaturated aliphatic, and aromatic structures. Above 350 °C, only bands attributed to carbon dioxide, carbon monoxide, methane (main peaks at 3016 cm^−1^ and 1304 cm^−1^) and small amounts of carbonyl compounds were detected. Further, the carbon yield of different starch aerogels, after carbonization at various temperatures, was estimated based on the results of EGA(FTIR)-TGA experiments and are gathered in Table 1.

The obtained values of carbon yields suggest that the carbon content in aerogel samples increases in the following order: rice starch < potato starch < maize starch. Additionally, it can be observed that the carbon yields for the samples of one type of starch increase along with the carbonization temperature decline.

**Figure 3:**
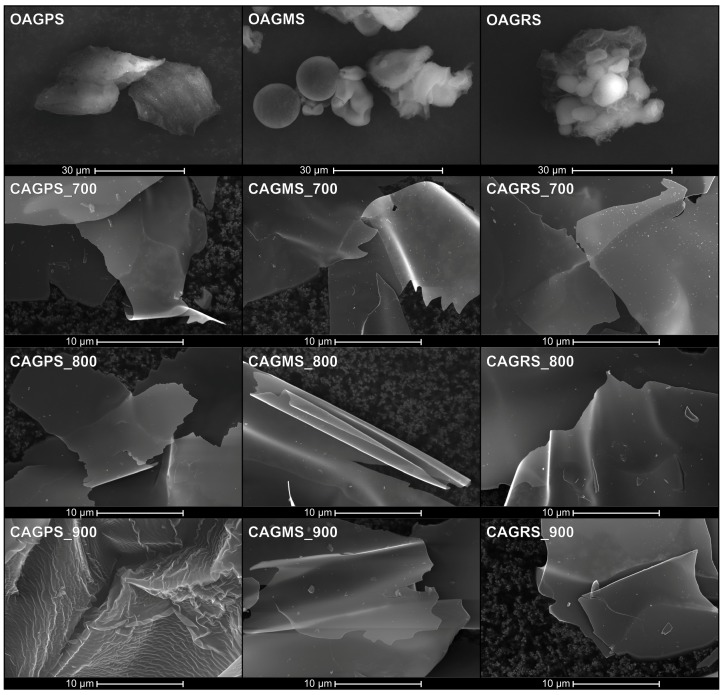
SEM micrographs of organic and carbon aerogels (CAGs) pyrolysed at 700 °C, 800 °C, and 900 °C obtained from different starches (PS: potato starch, MS: maize starch, RS: rice starch).

Figure 3 presents SEM micrographs of both organic and carbon aerogels obtained from different starches. It can be seen that organic aerogels are highly porous with soft, fluffy edges. The fraction of round grains can be observed in the samples obtained from maize and rice starch. Micrographs of CAGs samples after carbonization at high temperatures reveal similarity to highly graphitized hard carbon rather than to pyrolytic amorphous carbon materials. Obtained carbons have the form of ultra-thin, wrinkled sheets. The cracks and irregular, sharp edges are the result of the CAGs grinding process. Small particles visible on the micrographs backgrounds come from the carbon tape which was used to attach the organic and carbon aerogels powders to the sample holder.

**Figure 4:**
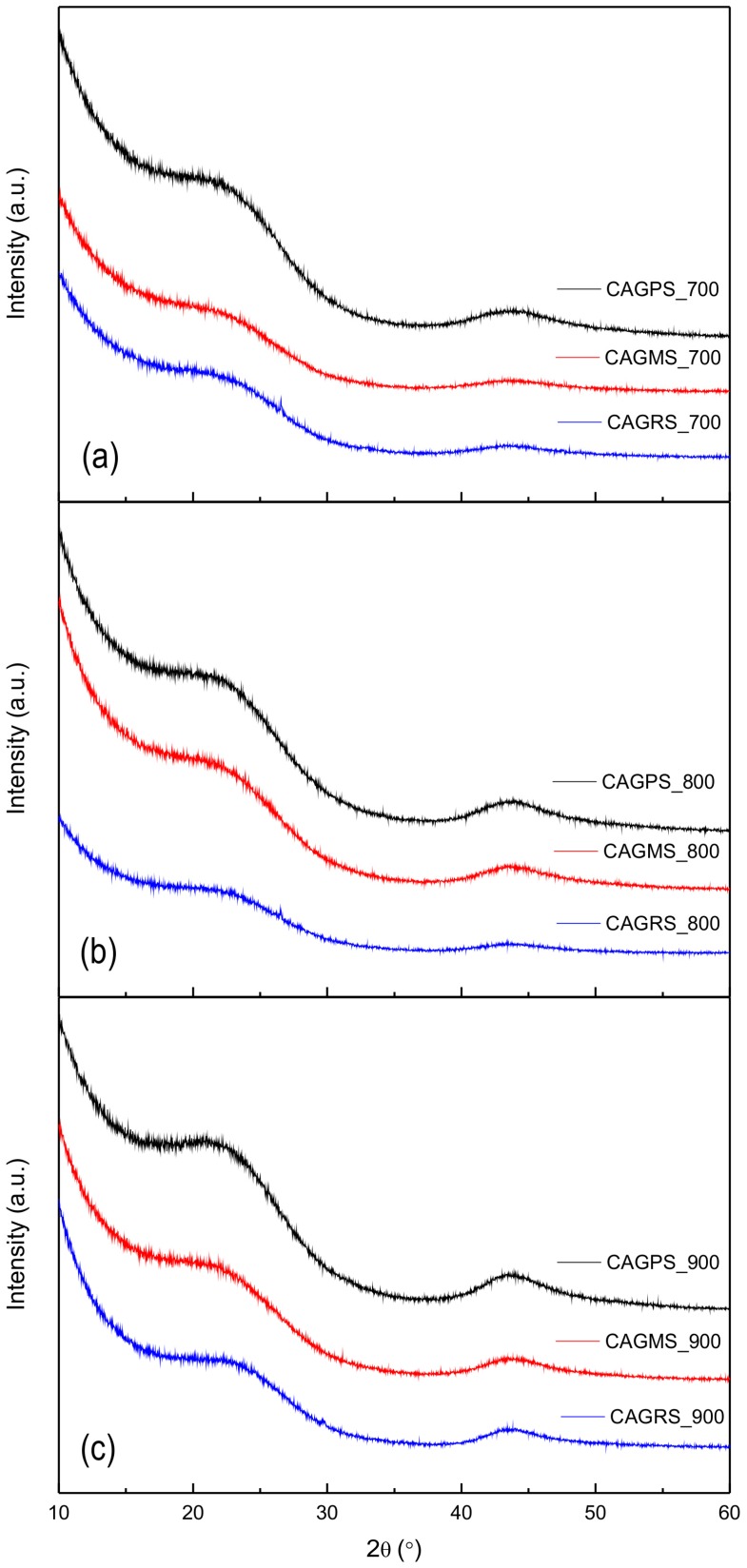
X-ray diffraction patterns of carbon aerogels pyrolysed at (**a**) 700 °C; (**b**) 800 °C and (**c**) 900 °C.

The X-ray diffraction patterns of carbon aerogels obtained from various types of starches, pyrolysed at 700 °C, 800 °C, and 900 °C, are shown in Figure 4a–c, respectively.

For all diffractograms, we can observe two broad humps at about 23° and 43° 2θ. These reflections can be ascribed to the graphene-like domains [36,37,38]. Along with the increase of the carbonization temperature, the intensity of aforementioned reflexes rises, which relates to a higher degree of carbon aerogels’ graphitization. Moreover, on the basis of these results, it can be concluded that potato starch-based carbon aerogels contain a greater amount of graphene-like domains regardless of the pyrolysis temperature.

The CHN analysis enabled the determination of carbon, hydrogen, and nitrogen content in carbon aerogels (Table 2).

The balance of the elemental composition was assumed to be oxygen. As it is observed, the amount of hydrogen decreased along with the increase of pyrolysis temperature for all carbon materials. It was also established that the weight of carbon in CAG samples increased with increasing pyrolysis temperature, while the amount of oxygen diminished. Some small deviations from this relation can be noted, but only for CAGMS material. On the contrary, the elemental composition of nitrogen did not change in a specific way with the carbonization temperature. All things considered, the best outgassing and the highest carbon composition (by weight) can be attributed to the rice starch-based carbon aerogel pyrolysed at 900 °C.

**Figure 5:**
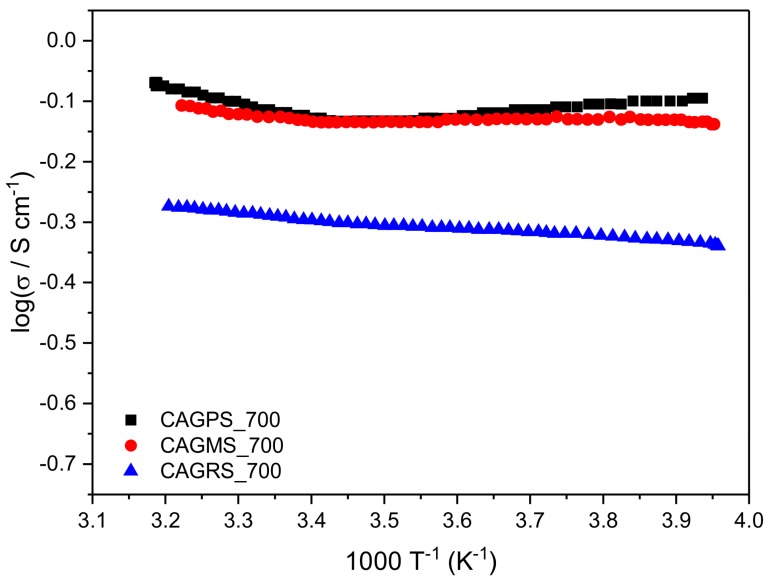
The Arrhenius plots of CAGPS, CAGMS, and CAGRS materials carbonized at 700 °C.

The electrical properties of obtained carbon aerogels were studied to verify the graphitization degree of these materials. All of the samples reveal very good electrical conductivity along with the very low activation energy of EC. The conductivity is directly connected with the amount of graphite-like domains. For highly graphitized carbons (samples pyrolysed at T ≥800 °C), the EC was above the measurability range of the used apparatus. That is why the Arrhenius plots of conductivity are illustrated only for materials carbonized at 700 °C (Figure 5).

Nevertheless, the presented results clearly show the difference between the samples. The electrical conductivity complies with the Arrhenius law $\sigma =\sigma_{0}\;\exp\left(\frac{-E_{a}}{k_{B}T}\right)$, where $\sigma_{0}$ is the pre-exponential factor, $E_{a}$ is the activation energy, and $k_{B}$ is the Boltzmann constant. The slope of the plot in the logσ vs. 1000∙T^−1^ coordinates enabled the evaluation of the activation energy. The calculated average values of $E_{a}$ in the range of −20 to +40 °C as well as σ estimated at 25 °C are gathered in Table 3.

As it can be noticed, all samples reveal very low activation energy, which may confirm the graphite-like structure of obtained carbons [39]. CAGPS_700 exhibits the highest σ value, which corresponds well with XRD data and proves the highest graphitization rate of potato starch-based samples.

**Figure 6:**
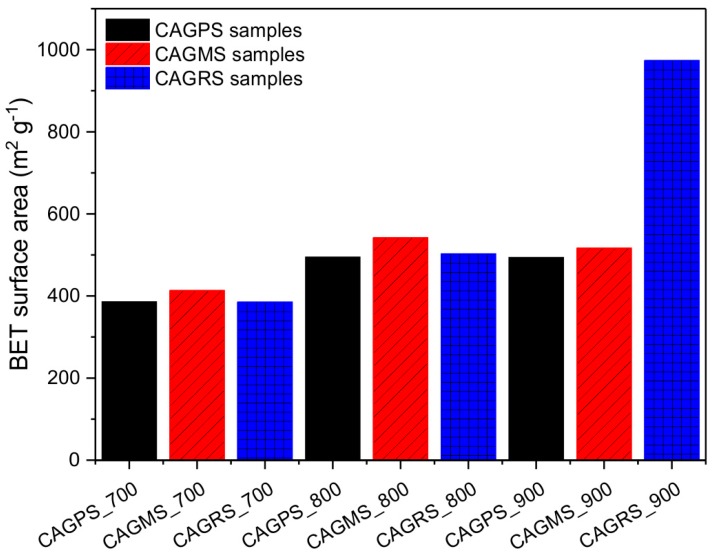
BET surface area of carbon aerogels.

The calculated values of BET (Brunauer-Emmett-Teller) surface area of obtained carbon aerogels are presented in Figure 6.

**Figure 7:**
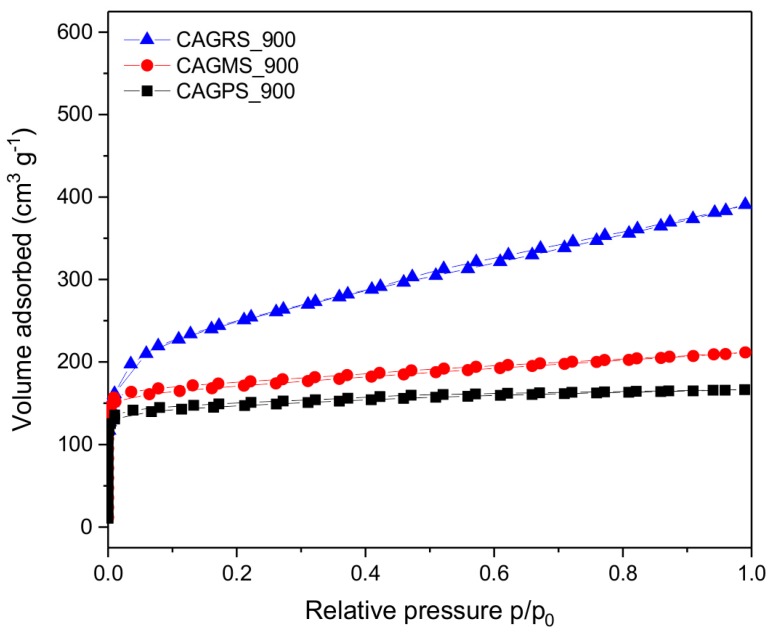
Nitrogen sorption isotherms of CAGPS, CAGMS, and CAGRS carbonized at 900 °C.

It can be observed that the specific surface area increases with higher pyrolysis temperature. With the increase of the pyrolysis temperature from 700 to 800 °C, the surface area of the samples increased from about 400 m^2^∙g^−1^ to about 500 m^2^∙g^−1^. Further increase of the pyrolysis temperature to 900 °C did not significantly influence the BET surface area of the samples. Only in the case of CAGRS_900, obtained from rice starch a great development of BET surface area to about 970 m^2^∙g^−1^ can be noted. The exemplary nitrogen adsorption–desorption isotherms for the samples carbonized at 900 °C are presented in Figure 7. In case of CAGPS and CAGMS samples, the isotherms of type I (a) (according to the IUPAC classification) [40] were obtained. As for the CAGRS sample, its isotherm was classified as I (b) type. Both these types of isotherms are characteristic of microporous solids with relatively small external surface, like activated carbons. In addition, the sharp uptake at very low partial pressures, together with lack of any hysteresis loop at $\frac{p}{p_{0}}>0.4$ proved the microporous nature of obtained carbon aerogels. On the whole, the porous characteristic of CAGs obtained in this study is in good accordance with the literature data relating to the carbon materials based on renewable organic sources [41,42]. Nonetheless, comparing with other samples, the CAGs indicate lower values of BET surface area.

## 4. Conclusions

The facile fabrication method of carbon aerogels in which alcogels were dried under ambient pressure in air atmosphere was presented. Using this method, carbon aerogels from three different starches (potato, maize, and rice) were obtained at different temperatures (700 °C, 800 °C, 900 °C). The complete analysis of the carbonization process of different starch aerogels performed using EGA(FTIR)-TGA showed great similarities in carbonization processes for studied starches. Despite the similarity in the pyrolysis processes, the obtained carbon aerogels feature different morphology and electrical conductivity. Low-temperature carbons (up to 800 °C) derived from maize precursor showed the highest specific surface areas, but for carbonization conducted at higher temperature, the largest surface development is observed for aerogels from rice precursor. The shape of the nitrogen sorption isotherms proved practically a purely microporous character of the samples. The electrical conductivity measurements suggest that carbon aerogels with the best electrical properties can be obtained from potato starch. All things considered, the obtained carbon materials—due to their developed surface area and high electrical conductivity—represent very attractive materials for electrochemical applications, especially for the energy storage systems.

## Figures and Tables

**Table 1:** Carbon yield after carbonization of aerogels at various temperatures based on thermogravimetric analysis coupled with evolved gas analysis with infrared spectroscopy detection (EGA(FTIR)-TGA) results.

Sample	Starch Type	Carbon Yield (wt %)		
		700 °C	800 °C	900 °C
OAGPS	potato starch	16.1	15.2	13.9
OAGMS	maize starch	17.1	16.2	14.8
OAGRS	rice starch	15.8	14.6	13.3

**Table 2:** The results of elemental analysis of carbon aerogels.

Sample	C (wt %)	H (wt %)	N (wt %)	O (wt %)
CAGPS_700	91.8	1.7	0.4	6.1
CAGPS_800	92.1	1.4	0.4	6.1
CAGPS_900	92.5	1.0	0.6	5.9
CAGMS_700	91.8	1.7	0.3	6.2
CAGMS_800	90.9	1.3	0.6	7.2
CAGMS_900	92.3	1.1	0.3	7.3
CAGRS_700	86.8	1.9	2.0	9.3
CAGRS_800	88.6	1.5	1.3	8.6
CAGRS_900	93.4	1.1	0.4	5.1

**Table 3:** Electrical properties of carbon aerogels pyrolysed at 700 °C.

Sample	*E_a_* (eV)	*σ* at ~25 °C (S∙cm^−1^)
CAGPS_700	0.007	0.833
CAGMS_700	0.004	0.751
CAGRS_700	0.015	0.513
